# Providing Web-Based Feedback and Social Norms Information to Reduce Student Alcohol Intake: A Multisite Investigation

**DOI:** 10.2196/jmir.1461

**Published:** 2010-12-19

**Authors:** Bridgette M Bewick, Robert West, Jan Gill, Fiona O'May, Brendan Mulhern, Michael Barkham, Andrew J Hill

**Affiliations:** ^4^Centre for Psychological Services ResearchUniversity of SheffieldWestern BankUnited Kingdom; ^3^School of Health SciencesQueen Margaret UniversityEdinburghUnited Kingdom; ^2^Division of BiostatisticsUniversity of LeedsLeedsUnited Kingdom; ^1^Academic Unit of Psychiatry and Behavioural SciencesLeeds Institute of Health SciencesUniversity of LeedsLeedsUnited Kingdom

**Keywords:** Student, eHealth, brief intervention, alcohol, Web-based interventions

## Abstract

**Background:**

Unhealthy alcohol use among university students is cause for concern, yet the level of help seeking behavior for alcohol use is low within the student population. Electronic brief interventions delivered via the Internet present an alternative to traditional treatments and could enable the delivery of interventions on a population basis. Further evidence is needed of the effectiveness of Internet-delivered interventions and of their generalizability across educational institutions.

**Objective:**

Our objective was to evaluate the effectiveness across 4 UK universities of a Web-based intervention for student alcohol use.

**Methods:**

In total, 1112 participants took part. Participants were stratified by educational institution, gender, age group, year of study, and self-reported weekly consumption of alcohol and randomly assigned to either the control arm or to the immediate or delayed intervention arms. Intervention participants gained access to the intervention between weeks 1 to 7 or weeks 8 to 15, respectively. The intervention provided electronic personalized feedback and social norms information on drinking behavior accessed by logging on to a website. Participants registered interest by completing a brief screening questionnaire and were then asked to complete 4 further assessments across the 24 weeks of the study. Assessments included a retrospective weekly drinking diary, the Alcohol Use Disorders Identification Test (AUDIT), and a readiness-to-change algorithm. The outcome variable was the number of units of alcohol consumed in the last week. The effect of treatment arm and time on units consumed last week and average units consumed per drinking occasion were investigated using repeated measures multivariate analysis of covariance (MANCOVA). In addition, the data were modeled using a longitudinal regression with time points clustered within students.

**Results:**

MANCOVA revealed a main effect of time on units of alcohol consumed over the last week. A longitudinal regression model showed an effect of assessment across time predicting that participants who completed at least 2 assessments reduced their drinking. The model predicted an additional effect of being assigned to an intervention arm, an effect that increased across time. Regression analysis predicted that being male or being assigned to an intervention arm increased the odds of not completing all assessments. The number of units of alcohol consumed over the last week at registration, age, university educational institution, and readiness to change were not predictive of completion.

**Conclusions:**

Delivering an electronic personalized feedback intervention to students via the Internet can be effective in reducing weekly alcohol consumption. The effect does not appear to differ by educational institution. Our model suggested that monitoring alone is likely to reduce weekly consumption over 24 weeks but that consumption could be further reduced by providing access to a Web-based intervention. Further research is needed to understand the apparent therapeutic effect of monitoring and how this can be utilized to enhance the effectiveness of brief Web-based interventions.

## Introduction

Unhealthy alcohol use among university students continues to be of concern given the immediate and long-term physical, psychological, and social consequences of this behavior [1-4]. The level of heavy episodic or binge drinking within this population [5] increases the risk of students engaging in risky, illegal, and violent behaviors [6-8]. In addition to the immediate personal and societal costs associated with alcohol misuse, heavy consumption during university is predictive of alcohol misuse and dependence in later life. Furthermore, help-seeking for alcohol problems is low [9], meaning that relatively few students access the traditional support services available.

Alcohol interventions using the Internet are being developed that build on the established brief interventions evidence base. These interventions are viewed as having potential benefit to those who have not sought traditional modes of support or treatment [10]. In addition, e-delivery may aid early self-identification of alcohol problems via the wide-scale access students have to the Internet. This combined with ability to enable confidential access at a time convenient to the user make this mode of delivery especially attractive.

Recent systematic reviews of electronic forms of alcohol intervention have indentified 17 randomized controlled trials involving young people [11,12]. A recent meta-analysis concluded that single sessions of personalized feedback, including those delivered electronically (without therapist input), can be effective in reducing problem drinking in the short-term (with follow-up up to 9 months after the intervention) [13]. Reviews suggest that interventions providing personalized feedback and social norms information can be effective [14]. Inconsistencies in outcome relate to weaknesses in the methodological quality of some evaluations [11,13-15], although over time there appears to have been a marked improvement in the quality of studies. In particular, there has been an increase in the number of studies using a randomized controlled (RCT) design and well-validated measures of alcohol consumption. Despite these advancements, published results from European trials investigating the effectiveness of Web-based interventions are relatively rare (in comparison with the number of trials published from North America and Australia). In addition, few trials explore the generalizability of effectiveness of interventions at multiple institutions. Indeed all the trials identified in a recent Cochrane review [14] were carried out at single educational institutions. Thus, there is a need to investigate if an intervention developed at one educational institution can be effective at modifying behavior of students based at other educational institutions (without the need for in-person contact during recruitment, assessment, or intervention delivery).

Our recent randomized controlled trial suggested that Web-based interventions for students, incorporating brief personalized feedback and social norms information, can be effective in reducing per occasion alcohol consumption among UK students [16]. The results showed that an electronic approach to delivering personalized feedback and social norms information could be effective in a European population. A second and larger RCT has replicated these findings and showed the reduction to be maintained at the 4-month follow-up. A limitation common to both these studies was the recruitment of students from only one educational institution. The existing evidence base says little about whether an intervention developed at one university will generalize to other educational institutions. Thus, the current research aimed to evaluate the effectiveness of a Web-based intervention developed at one educational institution for moderating the alcohol consumption of students from other educational institutions by including participants from 4 other UK universities. There is also little information on whether time of academic year affects outcome. Within the current research, it was hypothesized that the presentation of electronic personalized feedback and social norms information would reduce alcohol intake regardless of when during the academic year the intervention was available.

## Methods

### Procedure

The mode of student recruitment varied across educational institution; however, all used some form of e-recruitment (eg, notification included within weekly student union bulletin, posting of invitation within student portal, and direct email to students). In addition, some educational institutions posted paper-based posters around campus and provided verbal reminders to their students (eg, during induction seminars).

Interested students were invited to register during October 2007 (Time 0). The trial was conducted over a 27-week period beginning in November 2007 (Time 1).

Students who registered were asked to complete a retrospective drinking diary and the Alcohol Use Disorders Identification Test (AUDIT); these assessments are described in more detail below. At Time 0, students were also asked to provide details of their demographics including age, gender, ethnicity, graduate status, and area of study.

Only students who were consumers of alcohol and provided details of their alcohol consumption at Time 0 were eligible to enter the current study. All students who consumed alcohol at least once every 6 months were included in the current study. This was deemed important because (1) if found to be effective, the intervention would be made available to all students, and, therefore, an evaluation of the effect on those consuming below hazardous or harmful levels is required as it is possible such information could have an adverse impact, and (2) previous research has shown that misperceptions of social norms are not only the domain of those engaging in risky behavior, and, therefore, to correct misperceptions across the population, feedback was necessary for all students.

Eligible students were stratified by educational institution, gender, age group (above or below 21 at entry to undergraduate degree program), graduate student status, and self-reported weekly consumption of alcohol (within or above sensible drinking guidelines).

### Research Design

The study was an RCT with 3 arms: 1 control arm (assessment only) and 2 intervention arms (immediate and delayed access). Participants were stratified by educational institution, gender, age group, year of study, and self-reported weekly consumption of alcohol and randomly assigned to either the control or to the immediate or delayed intervention arms. Participants were not blind to their allocation.

After allocation to study arm, participants were emailed further information about the study along with an electronic link to the initial Time 1 (week 1) assessment. All contact with participants was via email. The Time 1 email contained an embedded link to either the control assessment electronic survey (completed by those in the control arm and in the delayed intervention arm) or to the intervention website (for those in the immediate intervention arm). Both websites contained identical project information and assessments (question presentation was the same between the two websites). Before completing their assessment participants were provided with a study briefing and provided informed consent online. Participants were advised that their having completed the earlier registration assessment did not mean they had to consent to taking part in the trial. Once participants in the intervention arm had completed their assessment, they received brief personalized feedback and social norms information via the website. Participants who did not complete the Time 1 assessment during week 1 were sent weekly reminders for up to 3 weeks (or until they had completed the assessment).

Participants in the immediate and delayed intervention arms had access to the intervention during weeks 1 through 7 or weeks 8 through 15, respectively. Regardless of which arm they were allocated to, participants were assessed at 5 time points.

**Figure 1:**
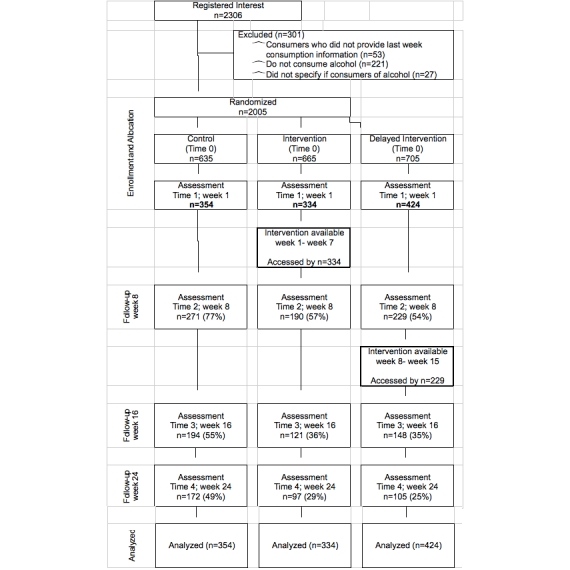
Participants flow through the trial

Following the initial assessment at Time 1, additional assessments were completed at week 8 (Time 2), week 16 (Time 3), and week 24 (Time 4) (see Figure 1). The follow-up assessments (at Times 2 through 4) for intervention participants were also collected via the control assessment electronic survey. Those in the delayed intervention arm completed their Time 2 assessment via the intervention website and received personalized feedback once their assessment was completed. Delayed intervention participants follow-up assessments (at Time 3 and Time 4) were completed via the control assessment electronic survey. On completion of each Time 1 through Time 4 assessment, participants were entered into a prize draw to win a £25 Amazon voucher (ie, 4 draws per educational institution).

The study was approved by the Leeds East National Health Service (NHS) Research Ethics Committee and, where required by education institutional procedures, also by the relevant university ethics committee.

### Sample Size

From previous work we ascertained that the average natural logarithm of the number of units of alcohol consumed over the last week plus 1 for students is approximately 1.3 with a standard deviation of 0.58 and, hence, a variance of 0.34. A change in natural logarithm of the number of units consumed over the last week plus 1 over the intervention period will therefore have a variance of less than 0.68 (ie, 2 times 0.34). We have taken it to be equal to 0.49 (ie, 0.7^2^).

The difference in the change in the natural logarithm of the number of units consumed over the last week plus 1 between two treatment arms might be tested with a *t* test where the relevant standard deviation is 1.3. A suitable difference in change in the natural logarithm of the number of units consumed over the last week plus 1 was taken as 0.5, so that we sought a standardized difference of 0.5. For a significance level of alpha equal to .05 and a power of 1 minus beta equal to .8, a sample size of 107 participants per treatment arm was required. Given the lower power of this test compared with the analysis we proposed, this set an upper limit to the sample size as 107 participants per treatment arm or 321 participants completing the trial. To allow for attrition we aimed to recruit at least 500 participants in total.

### Assessments

Participants were asked to complete a range of assessments detailing their alcohol consumption, level of alcohol dependence, readiness to change, psychological well-being and risk-taking behavior. Only those assessments of relevance to the reported analyses are described here.

Participants completed a 7-day retrospective drinking diary for the previous week at each of the 5 assessments at Time 0 through Time 4. To facilitate accuracy, participants were provided with a list of a variety of types of drinks (eg,175 milliliters red wine, 1 pint of ordinary strength lager) and asked to indicate how many of each they had consumed each day for the last 7 days. The number of drinks was then converted to standard UK units of alcohol (1 unit = 8 grams of pure ethanol) using published UK government guidelines. This retrospective drinking diary method has been previously used within a student population [16], and the approach has been recommended as a measure for groups that drink regularly [17]. The main outcome measure was total units consumed over the last week. A secondary outcome measure was the number of units consumed per average drinking occasion.

Participants completed the AUDIT at Times 0 through 4. AUDIT is a 10-item measure investigating the quantity and frequency of alcohol consumption, problems related to use, and dependence symptoms. Items are scored on a scale of 0 to 4, and a cutoff score of 8 is recommended for the identification of possible hazardous or harmful drinking [18,19]. The cross-national validation study of the AUDIT found high levels of sensitivity (.92) and specificity (.94) [20], and the measure has been widely used.

Readiness to change drinking behavior was measured (at Times 1 through 4) using a 3-question algorithm developed by Epler et al [21]. The questions investigate level of change in drinking (“Has the amount you drink changed in the past three months?”), propensity toward changing drinking behavior (“Are you interested in drinking less?”), and whether the perceived drinking level is too high (“Do you drink more than you should?”) The algorithm categorizes drinkers into a precontemplation group, a contemplation group, or an action group.

### Intervention

Participants in the immediate and delayed intervention arms gained access to Unitcheck (www.unitcheck.co.uk). Unitcheck provides personalized feedback on alcohol consumption and social norms information. This feedback was available every time participants visited the website and completed the online survey. The online feedback, delivered after students completed the retrospective drinking diary alongside a number of other questions outlined below, consisted of 3 main sections: (1) feedback on level of alcohol consumption, (2) social norms information, and (3) generic information.

### Feedback on Level of Alcohol Consumption

Participants were presented with statements indicating the number of alcohol units they consumed per week and the associated level of health risk. Statements were standardized for each risk level and gave advice about whether personal alcohol consumption should be reduced or maintained within the current sensible levels [22]. The number of alcohol-free days was indicated alongside information stating that it is advisable to have at least 2 per week. In addition, students who consumed at least twice the daily units recommended by the UK government (ie, females who consumed 6 or more units [48 grams pure ethanol] or males who consumed 8 or more units [64 grams pure ethanol]) were advised on the number of binge episodes during the week, and it was suggested that they may want to reduce the amount they consumed per occasion.

### Social Norms Information

Personalized statements were presented that summarized the percentage of university students who report drinking less alcohol than they consume. This was calculated relative to the risk level generated in section 1 of the feedback. The frequency of students within each risk level was established from data collected as part of an earlier university survey [23] and checked against the levels of consumption reported within the current sample. Information was also provided about the negative effects of alcohol intake reported by students who consume alcohol within the same risk category.

### Generic Information

Generic information provided standard advice on calculating units and the general health risks of high levels of consumption and outlined sensible drinking guidelines publicized in the United Kingdom. Tips for sensible drinking and the contact details of both local and national support services were also presented.

### Data Analysis

The effect of treatment arm and time on units consumed the previous week and average units consumed per drinking occasion were investigated using repeated measures multivariate analysis of covariance (general linear model) (MANCOVA). Within this analysis, the dependent variable was units consumed, the independent variable was treatment arm, and the covariate was the natural logarithm of the number of units consumed the previous week at registration. All analyses were carried out on the basis of condition allocation with the last known value brought forward. Due to the data being positively skewed, the natural logarithm of 1 plus the number of units consumed was used for all analyses. The number of units presented within the text and tables is the number of original scale units. These analyses were undertaken using SPSS, version 15 (SPSS Inc, Chicago, IL, USA).

Previous research has suggested differential attrition according to treatment arm and some trials have observed relatively high rates of attrition. These trial characteristics render the traditional repeated measures MANCOVA problematic, specifically liable to dropout bias. Therefore, an additional analysis of the primary outcome data (ie, units consumed the previous week) was planned that could accommodate these characteristics [24], namely, modeling the data using a longitudinal regression with time points clustered within students. That is, regression of the natural logarithm of the number of units plus 1 regressed upon occasion, male gender, age, and the other covariates considered. Measurements were clustered within individuals, making this a multilevel model. The model was fitted on a log scale, and this was inverted to present on the original scale of units. It was also possible that any observed effect of intervention could have been artificially produced by differential dropout, for example, heavier drinkers may have been less likely to complete assessments. To investigate this, a logistic regression was fitted to predict which students would not complete the study. Included in the regression analysis were age, educational institution, units consumed the previous week at Time 0, sex, condition arm, and readiness to change. To clarify the position with respect to previous alcohol consumption, a box plot was generated to compare noncompleters with assessment completers. This analysis was undertaken with Stata, version 11.0 (StataCorp, College Station, TX, USA).

## Results

A total of 2306 students registered interest in being involved in the project. Of these, 2005 eligible students were randomized to a study arm and invited to take part (see Figure 1 for exclusion criteria). Of these, 54% (1112/2005) of students provided informed consent to be involved in the trial, a valid username, and completed the Time 1 assessment. Of the 1112 students who completed the Time 1 assessment, 690 (62%) provided Time 2 data at week 8, 463 (42%) at Time 3 at week 16, and 374 (34%) provided Time 4 data at week 24 (see Figure 1).

Of the 1112 students enrolled in the study, 816 (73%) were female. Participants’ age ranged from 18 to 67 years (mean 21.45 yrs, SD 5.19). The majority of participants (95%) were undergraduate students, and 92% were predominately white/white British, based on self-reported choice from among several categories of ethnicity. Participants came from a range of subject areas, the largest of which were: medicine and health (319/1112 or 29%), sciences (175/1112 or 16%), arts (155/1112 or 14%), and social sciences (140/1112 or 13%). In all, 4 universities (3 English and 1 Scottish) took part in the study. The 4 universities were heterogeneous in terms of student populations (ie, the number of students potentially being invited to participate varied by educational institution: university A, approximately 15,000; university B, approximately 26,000; university C, approximately 5500; and university D, approximately 5000). The majority of participating students came from university A (591/1112 or 53%), followed by university B (344/1112 or 31%), university C (106/1112 or 10%), and university D (71/1112 or 6%).

Of the 1112 students enrolled in the study, 57% (637) scored 8 or more on the AUDIT and, therefore, their drinking could be considered potentially hazardous or harmful. At registration, the mean number of units of alcohol consumed over the previous week was 23.2 (SD 25.5). The majority of participants (829/1112 or 75%) reported consuming alcohol at least once a week. A further 15% (169/1112) reported fortnightly consumption with 10% (114/1112) consuming either once a month (n=80) or once every 6 months (n=34). Regarding readiness to change, at Time 1, a quarter of participants (276/1112 or 25%) were in the precontemplation phase, almost one-third (319/1112 or 29%) were in the contemplation phase, and approximately one-fifth (246/1112 or 22%) were in the action phase. The remaining participants had either missing data (196/1112 or 18%) or had at that time identified themselves as nondrinkers (75/1112 or 7%). The demographics of participants by treatment arm are provided in Table 1.

**Table 1:** Demographics of participants at baseline by treatment arm allocation

		Treatment arm			
		Assessment only n = 354	Delayed access n = 424	Immediate access n = 334	Total n = 1112
Female, n (%)		265 (75%)	304 (72%)	247 (74%)	816 (73%)
Age, mean (SD)		21.3 (4.6)	21.6 (5.8)	21.4 (5.1)	21.5 (5.2)
Undergraduate, n (%)		336 (95%)	411 (97%)	313 (94%)	1060 (95%)
White/white British, n (%)		329 (93%)	388 (92%)	301 (90%)	1018 (92%)
**Subject area**						
	Medicine and health, n (%)	106 (30%)	115 (27%)	98 (29%)	319 (29%)	
	Arts, n (%)	44 (13%)	66 (16%)	45 (14%)	155 (14%)	
	Social sciences, n (%)	46 (13%)	50 (12%)	44 (13%)	140 (13%)	
**Educational institution**						
	A, n (%)	177 (50%)	228 (54%)	186 (56%)	591 (53%)	
	B, n (%)	118 (33%)	125 (30%)	101 (30%)	344 (31%)	
	C, n (%)	36 (10%)	42 (10%)	28 (8%)	106 (10%)	
	D, n (%)	23 (7%)	29 (7%)	19 (6%)	74 (6%)	

Alcohol consumed over the last week and per average occasion is shown in Table 2. Repeated measures MANCOVA revealed a main effect of time on units consumed over the previous week (F_3,3324_ = 6.42, *P* < .01). Pairwise comparisons showed a significant decrease between Time 1 and all other time points (ie, Time 2 to Time 4) but no significant differences between Time 2, Time 3, or Time 4. There was however no significant time by treatment arm interaction (F_6,3324_ = 1.30, *P* = .24).

Repeated measures MANCOVA revealed no main effect of time on average units consumed per drinking occasion over the previous week (F_3,3324_ = 0.53, *P* = .67). There was a significant time by treatment arm interaction (F_6,3324_ = 2.85 *P* < .001). Further analysis revealed a significant time by consumption effect for the control arm (F_3,1059_ = 12.08, *P* < .01) with significant reductions between Time 1 and Time 2 (*P <* .01) and between Time 1 and Time 3 and between Time 1 and Time 4 (*P* < .01). There was also a significant time effect for the delayed intervention arm (F_3,1269_ = 11.46, *P* < .01) with significant reductions between Time 1 and Time 2, Time 1 and Time 3, and Time 1 and Time 4 (*P* < .01). No significant time effect was observed in the immediate intervention arm (F_3,999_ = 0.53, *P =* .66).

**Table 2:** Mean (SD) reported units consumed in the previous week by treatment arm and average per occasion over time

			Time 0	Time 1	Time 2	Time 3	Time 4
		n	Mean (SD)	Mean (SD)	Mean (SD)	Mean (SD)	Mean (SD)
**Units consumed over theprevious week**							
	Control	354	23.5 (24.0)	17.5 (27.4)	13.6 (19.8)	14.6 (19.6)	15.0 (20.7)
	Delayed intervention	424	23.5 (26.1)	14.7 (18.8)	13.4 (18.6)	12.8 (17.9)	11.9 (17.4)
	Intervention	334	22.6 (26.4)	15.2 (20.0)	14.5 (20.2)	14.2 (21.1)	13.9 (21.7)
	TOTAL	1112	23.2 (25.5)	15.8 (22.3)	13.8 (19.5)	13.8 (19.5)	13.5 (19.8)
**Average units consumed per drinking occasion over the last week**							
	Control	354	14.3 (11.2)	11.0 (8.8)	9.5 (8.5)	9.7 (11.0)	9.3 (11.1)
	Delayed intervention	424	14.2(12.9)	10.2 (8.6)	9.3 (9.0)	8.8 (8.3)	8.9 (9.0)
	Intervention	334	13.7 (12.3)	8.9 (8.3)	9.1 (10.8)	9.3 (11.1)	9.0 (11.2)
	TOTAL	1112	14.1 (12.2)	10.1 (8.6)	9.3 (9.4)	9.2 (10.1)	9.1 (10.3)

Included in the longitudinal regression model were monitoring status (ie, if participants completed any assessment Time 1 through Time 4), sex, and treatment arm (dichotomized to treatment/no treatment), and time since treatment (irrespective of time of initial access to the intervention). Readiness to change, if access to the intervention was immediate or delayed, and level of consumption at Time 0 were all excluded from the final model as they did not add significantly to the prediction. The longitudinal regression model showed a significant effect of assessment (without intervention) on change across time, showing that participants who completed at least two assessments reduced their drinking (Table 3). The model also predicted an additional effect of being assigned to 1 of the 2 intervention arms, an effect that increased across time.

The model predicted that at week 24 without any assessment completion (ie. completing only Time 0 assessment), females drank 11.5 units per week while males drank 16.0 units. As can be seen in Table 3, when students completed at least 2 of the 5 assessments, predicted consumption decreased to 6.1 units for females and 8.4 units for males. When assigned to an intervention arm, there was an additional effect that increased across time with the model predicting that at week 24 females in the intervention arm had reduced their previous week unit consumption to 3.7 and males in the intervention arm had reduced their previous week consumption to 5.2 units per week. Despite the variation in individual drinking patterns across time, the data included enough observations to see an effect of the intervention. Table 4 provides details of the regression coefficients fitted in the longitudinal model. In addition, an intercept term of 2.44 corresponded to the outcome, log (1 + units consumed), for a female participant at baseline. The model yielded an overall *R* *2* value of 0.06 and an interclass correlation coefficient of 0.14, indicating that there was significant variation between participants and over time. 

**Table 3:** Prediction of units consumed over the last week at each time point (longitudinal regression model)

	Units Consumed in the Previous Week	
	Females	Males
Without monitoring (ie, Time 0 assessment only)	11.5	16.0
With assessment completion but no intervention (ie, completed at least 2 of the 5 assessments)	6.1	8.4
Assigned to an intervention arm, consumption at week 8 post intervention delivery	5.5	7.6
Assigned to an intervention arm, consumption at week 16 post intervention delivery	5.1	7.1
Assigned to an intervention arm, consumption at week 24 post intervention delivery	3.7	5.2

**Table 4:** Table of coefficients for longitudinal regression model: log (1+units consumed over the last week) regressed on monitoring status, male sex, and duration since treatment (irrespective of when intervention was first delivered) by restricted maximum likelihood

Covariate		Coefficient	95% Confidence Interval	*P* Value
Monitored (ie, completed at least 2 of the 5 assessments)		-0.64	(-0.74 to -0.54)	< .001
Male		0.33	(0.22-0.43)	< .001
**Number of weeks after intervention**				
	8 weeks	- 0.12	(-0.29 to 0.04)	0.14
	16 weeks	- 0.17	(-0.36 to 0.03)	0.09
	24 weeks	- 0.54	(-0.83 to -0.26)	.001

**Figure 2:**
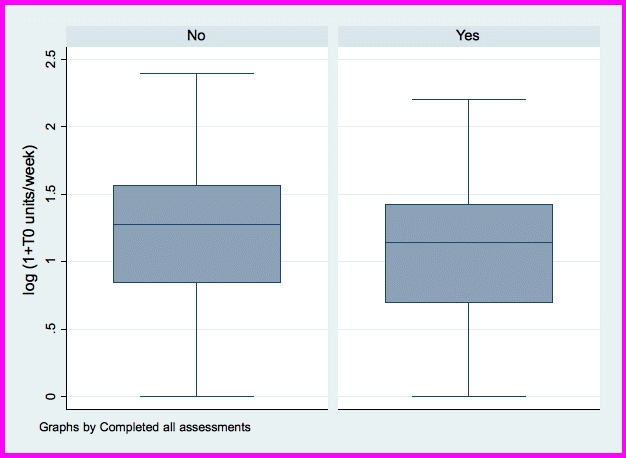
Average number of units of alcohol consumed over the previous week (expressed as the natural logarithm of the number of units plus 1) at baseline (Time 0) by assessment completion status

Regarding the possible effects of differential assessment completion, 26% (293/1112) of participants completed all 5 assessments with the remaining 74% (819/1112) of participants being classified as nonassessment completers (ie, completing between 2 and 4 assessments). Regression analysis showed that age, education institution, previous week unit consumption at Time 0, and readiness to change were unrelated to completion. The box plot summarizing units consumed over the previous week at Time 0 supported the regression analysis (Figure 2). The difference in the average number of alcohol units consumed in the previous week between those that completed all assessments and participants that did not is less than 0.4 units (with those who completed all assessments drinking slightly less). Being male or being assigned to the intervention increased the odds of not completing all assessments. Males had double the odds of not completing (odds ratio [OR] 2.10, 95% confidence interval [CI] 1.48-2.97) and those in the intervention had approximately triple the odds of not completing (immediate intervention arm OR 2.52, 95% CI 1.80-3.53; delayed intervention arm OR 3.47, 95% CI 2.49-4.85). The completion odds ratio was further supported by chi-square analysis that showed a significant association between the treatment arm and completion of all assessments (c^2^ = 67.4, *P* < .001; see Table 5). 

**Table 5:** Completion status by treatment arm

	Treatment Arm			
Completed All Assessments	Assessment Only n (%)	Delayed Access n (%)	Immediate Access n (%)	Total n (%)
Yes	150 (42%)	73 (17%)	74 (22%)	297 (27%)
No	204 (58%)	351(83%)	260 (78%)	815 (73%)
Totals	354 (100%)	424 (100%)	334 (100%)	1112 (100%)

## Discussion

### Principal Results

This study suggests that delivering an electronic brief intervention to students can be effective in reducing alcohol consumption. The results showed a significant effect of time and/or assessment completion. In addition, the intervention had an independent effect on reducing the number of units of alcohol consumed weekly. Neither educational institution nor time of academic year had an impact on intervention effectiveness. To the authors’ knowledge, these results are the first evaluation of an electronic Web-based feedback and social norms alcohol intervention to include students from multiple universities from outside of North America.

### Comparison With Prior Work

The current results are in line with previous research on electronic Web-based interventions that have reported significant decreases in total consumption per week [25-28]. In some of these studies, this decrease remained significant at the 3-month follow-up [25,26]. Unlike our earlier study [16], the current study found a significant reduction in units consumed per week where previously we found only an impact on units consumed per occasion. Within the current study, the regression analysis showed that males entered the study with a higher total number of units consumed over the last week. This finding is in agreement with other literature, which suggests that males consume more alcohol than females [23]. Being male increased the odds of not completing all assessments. Given that males have a tendency to consume higher levels of alcohol compared with females, further work is needed to understand how best to keep male participants engaged.

According to our analysis, assessment alone had a significant effect on drinking. It is, therefore, possible that completing an assessment led participants to monitor and reflect on their own behavior leading to a decrease in consumption. Such a decrease in consumption by assessed participants is apparent in the literature [29,30]. Our results suggest that this monitoring effect did not increase alongside the number of assessments, but that the completion of 2 or more assessments predicted a similar level of reduction.

Reactivity to assessment has been documented in the literature within randomized controlled trials [31-33] and within studies designed specifically to investigate reactivity [34,35]. It is possible that the reactivity effect is due to a reporting bias (eg, social desirability may mean that participants, consciously or subconsciously, report a change in behavior without any actual behavior change). Given that this is a limitation of all self-report data, the utility of such an argument is unclear. One alternative is to suggest that monitoring alone can lead to behavior change and, therefore, given that monitoring is likely to be carried out only in the context of potential treatment, monitoring can be viewed as one of the active components in brief interventions. It has previously been suggested that comparing intervention arms to assessment only may lead to the underestimation of the impact of brief interventions—especially given that individuals who are not seeking treatment are unlikely to have their behavior monitored outside of a randomized controlled trial [32]. Within the current study, being assigned to an intervention arm further increased the reduction in weekly consumption. This suggests that while monitoring alone appears to be effective in reducing consumption, this reduction can be further enhanced by the delivery of a brief personalized Web-based intervention.

The high proportion of students identified as drinking at potentially hazardous or harmful levels suggests that students engaging in unhealthy drinking behavior are interested in engaging with Web-based resources, and this is a strength of the current study. The ability to include such students is admirable given some studies have reported engaging a greater proportion of low-level consumers (eg, [36]). This is reinforced by the finding that assessment completion was not predicted by level of alcohol consumption, especially as previous research has reported higher levels of attrition among heavier consumers of alcohol [16,37]. In addition, it is noteworthy that 75% (563/1112) of participants included in the trial who were assigned to receive the intervention engaged with the Unitcheck website on at least 1 occasion. It is also encouraging that the time of academic year made no difference to outcome.

### Limitations

This study is the first outside of North America to engage students from multiple educational institutions and to include medium-term follow-up. A number of limitations need to be considered, however, when interpreting the results. First, although 75% of intervention participants accessed the intervention, the percentage that engaged with follow-up assessments was considerably lower, with only 26% (293/1112) completing all 5 assessments. Second, while participants from 4 educational institutions were involved, the relatively low number from some educational institutions highlights the difficulties in multisite interventions. In particular, it draws attention to the need to understand further how to engage students from universities that have not traditionally delivered health information to their students and who are perhaps not as receptive to receiving brief interventions via this medium. Third, while there was a 4-month follow-up period, these results say little about the longer term impact of the intervention. The long-term impact of electronic brief interventions is still uncertain. Nor is it understood how, if at all, repeated access to such interventions is likely to support behavior change. This is of particular importance to Web-based interventions that can be a repeated source of feedback to students at a time that they choose. It is a potential strength of e-interventions that students could be encouraged to repeatedly access feedback while incurring minimal additional costs. Fifth, the longitudinal regression analysis cannot determine if it is monitoring or willingness to be monitored that accounts for the reduction in consumption. The analysis attempted to account for this; however, we cannot rule out that willingness to be monitored (rather than engaging in the monitoring process itself) is an alternative explanation.

### Conclusions

This RCT confirms that Web-based interventions for alcohol can be effective in student samples. That the effect does not appear to vary across educational institutions is encouraging. It is hypothesized that differences in levels of recruitment across educational institutions are likely to be related to use of the Internet to deliver health information to students. Further work is needed, however, to understand how contextual factors can best be optimized in order to engage students. Within this study, much of the observed effect was apparently due to self-monitoring, but there was an additional effect of the intervention. Research is needed to understand how the individual elements of the personalized feedback interact with this self-monitoring effect in order to enhance the effectiveness of Web-based interventions.

## Abbreviations

AUDIT: Alcohol Use Disorders Identification Test
NHS: National Health Service
RCT: randomized controlled trial

## References

[1] Gill JS (2002). Reported levels of alcohol consumption and binge drinking within the UK undergraduate student population over the last 25 years. Alcohol Alcohol.

[2] Schulenberg JE, Maggs JL (2002). A developmental perspective on alcohol use and heavy drinking during adolescence and the transition to young adulthood. J Stud Alcohol Suppl.

[3] (2003). Royal College of Psychiatrists.

[4] Andersson A, Wiréhn AB, Olvander C, Ekman DS, Bendtsen P (2009). Alcohol use among university students in Sweden measured by an electronic screening instrument. BMC Public Health.

[5] White HR, Morgan TJ, Pugh LA, Celinska K, Labouvie EW, Pandina RJ (2006). Evaluating two brief substance-use interventions for mandated college students. J Stud Alcohol.

[6] Delk EW, Meilman PW (1996). Alcohol use among college students in Scotland compared with norms from the United States. J Am Coll Health.

[7] Orford J, Waller S, Peto J (1974). Drinking behavior and attitudes and their correlates among university students in England. I. Principal components in the drinking domain. II. Personality and social influence. III. Sex differences. Q J Stud Alcohol.

[8] Wechsler H, Davenport A, Dowdall G, Moeykens B, Castillo S (1994). Health and behavioral consequences of binge drinking in college. A national survey of students at 140 campuses. JAMA.

[9] Wechsler H, Lee JE, Kuo M, Seibring M, Nelson TF, Lee H (2002). Trends in college binge drinking during a period of increased prevention efforts. Findings from 4 Harvard School of Public Health College Alcohol Study surveys: 1993-2001. J Am Coll Health.

[10] Koski-Jänne A, Cunningham J (2001). Interest in different forms of self-help in a general population sample of drinkers. Addict Behav.

[11] Bewick BM, Trusler K, Barkham M, Hill AJ, Cahill J, Mulhern B (2008). The effectiveness of web-based interventions designed to decrease alcohol consumption--a systematic review. Prev Med.

[12] Cunningham JA, Khadjesari Z, Bewick BM, Riper H Internet-based interventions for problem drinkers: From efficacy trials to implementation. Drug Alcohol Rev.

[13] Elliott JC, Carey KB, Bolles JR (2008). Computer-based interventions for college drinking: a qualitative review. Addict Behav.

[14] Moreira MT, Smith LA, Foxcroft D (2009). Social norms interventions to reduce alcohol misuse in university or college students. Cochrane Database Syst Rev.

[15] Riper H, van Straten A, Keuken M, Smit F, Schippers G, Cuijpers P (2009). Curbing problem drinking with personalized-feedback interventions: a meta-analysis. Am J Prev Med.

[16] Bewick BM, Trusler K, Mulhern B, Barkham M, Hill AJ (2008). The feasibility and effectiveness of a web-based personalised feedback and social norms alcohol intervention in UK university students: a randomised control trial. Addict Behav.

[17] Dawson DA (2003). Methodological issues in measuring alcohol use. Alcohol Res Health.

[18] Babor TF, Higgins-Biddle JC, Saunders JB, Monteiro MG World Health Organization.

[19] Saunders J, Aasland OG, Babor T, de la Fuente JR, Grant M (1993). Development of the Alcohol Use Disorders Identification Test (AUDIT): WHO Collaborative Project on Early Detection of Persons with Harmful Alcohol Consumption--II. Addiction.

[20] Saunders JB, Aasland OG, Amundsen A, Grant M (1993). Alcohol consumption and related problems among primary health care patients: WHO collaborative project on early detection of persons with harmful alcohol consumption--I. Addiction.

[21] Epler AJ, Kivlahan DR, Bush KR, Dobie DJ, Bradley KA (2005). A brief readiness to change drinking algorithm: concurrent validity in female VA primary care patients. Addict Behav.

[22] Cabinet Office (2004). Prime Minister's Strategy Unit.

[23] Bewick BM, Mulhern B, Barkham M, Trusler K, Hill AJ, Stiles WB (2008). Changes in undergraduate student alcohol consumption as they progress through university. BMC Public Health.

[24] Laird NM, Ware JH (1982). Random-effects models for longitudinal data. Biometrics.

[25] Cunningham JA, Humphreys K, Koski-Jännes A, Cordingley J (2005). Internet and paper self-help materials for problem drinking: is there an additive effect?. Addict Behav.

[26] Kypri K, Langley JD, Saunders JB, Cashell-Smith ML, Herbison P (2008). Randomized controlled trial of web-based alcohol screening and brief intervention in primary care. Arch Intern Med.

[27] Walters ST, Vader AM, Harris R, Jouriles EN (2009). Reactivity to alcohol assessment measures: an experimental test. Addiction.

[28] Saitz R, Palfai TP, Freedner N, Winter MR, Macdonald A, Lu J, Ozonoff A, Rosenbloom DL, Dejong W (2007). Screening and brief intervention online for college students: the ihealth study. Alcohol Alcohol.

[29] Kypri K, Saunders JB, Williams SM, McGee RO, Langley JD, Cashell-Smith ML, Gallagher SJ (2004). Web-based screening and brief intervention for hazardous drinking: a double-blind randomized controlled trial. Addiction.

[30] Chiauzzi E, Green TG, Lord S, Thum C, Goldstein M (2005). My student body: a high-risk drinking prevention web site for college students. J Am Coll Health.

[31] WHO Brief Intervention Study Group (1996). A cross-national trial of brief interventions with heavy drinkers.. Am J Public Health.

[32] Fleming MF, Barry KL, Manwell LB, Johnson K, London R (1997). Brief physician advice for problem alcohol drinkers. A randomized controlled trial in community-based primary care practices. JAMA.

[33] Bien TH, Miller WR, Tonigan SJ (1993). Brief interventions for alcohol problems: a review. Addiction.

[34] Kypri K, Langley JD, Saunders JB, Cashell-Smith ML (2007). Assessment may conceal therapeutic benefit: findings from a randomized controlled trial for hazardous drinking. Addiction.

[35] McCambridge J, Day M (2008). Randomized controlled trial of the effects of completing the Alcohol Use Disorders Identification Test questionnaire on self-reported hazardous drinking. Addiction.

[36] Kypri K, McAnally HM (2005). Randomized controlled trial of a web-based primary care intervention for multiple health risk behaviors. Prev Med.

[37] Edwards AGK, Rollnick S (1997). Outcome studies of brief alcohol intervention in general practice: the problem of lost subjects. Addiction.

